# Plasma Levels of Mid-Regional Proadrenomedullin Accurately Identify H1N1pdm09 Influenza Virus Patients with Risk of Intensive Care Admission and Mortality in the Emergency Department

**DOI:** 10.3390/jpm12010084

**Published:** 2022-01-10

**Authors:** Blanca Valenzuela-Méndez, Francisco Valenzuela-Sánchez, Juan Francisco Rodríguez-Gutiérrez, Rafael Bohollo-de-Austria, Ángel Estella, Pilar Martínez-García, María Ángela González-García, Jordi Rello

**Affiliations:** 1 Gynecology and Obstetrics Department, Hospital Municipal de Badalona, Universitat Autonòma de Barcelona, 08193 Barcelona, Spain; blavalmen@gmail.com; 2 Critical Care Medicine Unit, University Hospital of Jerez, 11407 Jerez de la Frontera, Spain; rafabodea@yahoo.es (R.B.-d.-A.); litoestella@hotmail.com (Á.E.); 3 Hematology Department, University Hospital of Jerez, 11407 Jerez de la Frontera, Spain; kikorg1977@hotmail.com; 4 Department of Medicine Faculty, Medicine University of Cádiz, 11003 Cádiz, Spain; 5 Critical Care Medicine Unit, University Hospital Puerto Real, 11510 Puerto Real, Spain; pilar.martinezgarcia@gmail.com; 6 Department of Clinical Analysis, University Hospital of Jerez, 11407 Jerez de la Frontera, Spain; m.angela.gg@hotmail.com; 7 CHRU Nîmes, 30900 Nimes, France; 8 Valld’Hebron Institut of Research, CIBERES, 08035 Barcelona, Spain

**Keywords:** H1N1 influenza pneumonia, biomarkers, MR proadrenomedullin, procalcitonin, ferritin, CRP, sepsis, virus, ICU admission

## Abstract

Early identification of severe viral pneumonia in influenza virus A (H1N1pdm09) patients is extremely important for prompt admission to the ICU. The objective is to evaluate the usefulness of MR-proadrenomedullin (MR-proADM) compared to C reactive protein (CRP), procalcitonin (PCT), and ferritin in the prognosis of influenza A pneumonia. This prospective, observational, multicenter study included one hundred thirteen patients with confirmed influenza virus A (H1N1pdm09) admitted to an Emergency Department and ICUs of six hospitals in Spain. Measurements and Main Results: one-hundred thirteen patients with confirmed influenza virus A (H1N1pdm09) were enrolled. Seventy-five subjects (mortality 29.3%) with severe pneumonia caused by influenza A H1N1pdm09 virus (H1N1vIPN) were compared with 38 controls (CG).The median MR-proADM levels at hospital admission were 1.2 nmol/L (IQR (0.8–2.6) vs. 0.5 nmol/L (IQR 0.2–0.9) in the CG (*p* = 0.01), and PCT levels were 0.43 μg/L (IQR 0.2–1.2) in the H1N1vIPN group and 0.1 μg/L (IQR 0.1–0.2) in the CG (*p* < 0.01). CRP levels at admission were 15.5 mg/dL(IQR 9.2–24.9) in H1N1vIPN and 8.6 mg/dL(IQR 3–17.3) in the CG (*p* < 0.01). Ferritin levels at admission were 558.1 ng/mL(IQR 180–1880) in H1N1vIPN and 167.7 ng/mL(IQR 34.8–292.9) in the CG (*p* < 0.01). A breakpoint for hospital admission of MR-proADM of 1.1 nmol/L showed a sensitivity of 55% and a specificity of 90% (AUC-ROC0.822). Non-survivors showed higher MR-proADM levels: median of 2.5 nmol/L vs. 0.9 nmol/L among survivors (*p* < 0.01). PCT, CRP, and ferritin levels also showed significant differences in predicting mortality. The MR-proADM AUC-ROC for mortality was 0.853 (*p* < 0.01). In a Cox proportional hazards model, MR-proADM levels > 1.2 nmol/L at hospital admission were significant predictive factors for ICU and 90-day mortality (HR: 1.3). Conclusions: the initial MR-proADM, ferritin, CRP, and PCT levels effectively determine adverse outcomes and risk of ICU admission and mortality in patients with influenza virus pneumonia. MR-proADM has the highest potency for survival prediction.

## 1. Introduction

Compared to other types of pneumonia, patients with severe pneumonia caused by influenza A H1N1pdm09 virus (H1N1vIPN) have particular features of their own. The flu pandemic of 1918 [1] was characterized by increased mortality in young adults, obese patients, and pregnant women [2,3], a phenomenon that remains unexplained but was repeated in the pandemic of 2009. H1N1 influenza affects young patients and others with important comorbidities and is the main cause of viral community-acquired pneumonia. Morbidity and mortality are high, with a hospitalization rate of around 10%, of which 13–45% are admitted to the ICU; despite advances in treatment, mortality remains around 30% [4,5].

Early identification of severe viral pneumonia is critical because mortality is high, and predicting the severity is vital in order to allow prompt admission to the ICU. Community-acquired pneumonia-specific scores (PSI, CURB 65, or PIRO-CAP) have not proved helpful in these patients [6,7].

The APACHE II and SOFA scoring systems require recording the lowest values of various clinical and biological parameters within the first 24 h of hospitalization [8]. In contrast, biomarkers can be measured rapidly within hours of the admission of patients to the ER or ICU. PCR and PCT have been the most widely used without fully resolving, especially in patients with virus infections. CRP is a nonspecific marker of inflammation that is increased in other non-infectious diseases. PCT is considered a sensitive marker for bacterial infections and has been considered a useful biomarker for the diagnosis, prognosis, and monitoring of antibiotic treatment [9,10]. High levels of ferritin have been found in infectious diseases as an inflammatory marker or as part of the hemophagocytic syndrome (SHF). This syndrome has been frequently associated with viral infections [11].

The fundamental biological effects of adrenomedullin (ADM), a hormone widely distributed in tissues with a very short half-life, include vasodilator, positive inotropic, diuretic, natriuretic, bronchodilator, and bactericidal. Very high levels of ADM have been described in septic patients, interacting directly with the relaxation of vascular tone, triggering hypotension in these patients. MR-proADM is a fragment of 48 amino acids that splits from the final proADM molecule in a ratio of 1:1 with ADM [12]. Plasma MR-proADM levels, the stable part of the mid-region of proadrenomedullin, directly and proportionally show ADM levels and activity, and it has been stably detected in plasma and can be reliably determined in clinical practice. High levels of MR-proadrenomedullin (MR-proADM) have been described in critical sepsis patients. The increase is directly related to the relaxation of vascular tone and, therefore, hypotension and the presence of organ failure in patients with septic shock [13,14].

Saeed K et al. [15] recently published a large multicenter study showing the usefulness of this biomarker in initial medical care in the ED, in the evolution of patients and the progression of the disease. MR-proADM values below 0.87 nmol/L identified patients of low severity and who can be safely discharged without increasing readmissions and mortality. It also showed in the same study that MR proADM values greater than 1.54 nmol/L signified a high incidence of mortality and the need for admission to the ICU statistically independently. González del Castillo et al. [16] used the cut-off point previously observed [15] for hospital treatment (>0.87 nmol/L) or ambulatory (≤0.87 nmol/L). The result was a reduction in hospitalizations by 20% using MR-proADM levels, with similar safety. It concludes that implementing an MR-proADM algorithm optimizes emergency workflows efficiently and sustainably.

The influenza virus affects the respiratory tract directly, or through the inflammatory response it generates. The mechanisms involved, beyond a general inflammatory profile, are still unresolved. Multiple mechanisms have been described, including airway obstruction, loss of alveolar structure, loss of integrity of the lung epithelium due to direct epithelial cell death, and degradation of the endothelial glycocalyx (EG) [17]. Deterioration of the EG has been demonstrated in ARDS established by the influenza virus, and have an essential role in controlling vascular permeability and the adhesion and migration of neutrophils; both mechanisms play a well-known role in the pathophysiology of ARDS [18]. Microcirculation disorder has been demonstrated by a study of sublingual microcirculation, evaluated by near-infrared spectroscopy (NIRS). These data suggest that microcirculatory abnormalities are present in patients with acute lung injury associated with H1N1vIPN regardless of bacterial coinfection [19].

We hypothesize that MR-proADM plasma levels could predict disease severity in patients with influenza A virus pneumonia at the emergency room. The objective of this study is to determine the usefulness of MR-proADM in comparison with C-reactive protein (CRP), procalcitonin (PCT), and ferritin for anticipating ICU and 90-day mortality of patients with influenza A virus pneumonia. A secondary objective is to assess the power of these biomarkers to identify patients requiring ICU admission.

## 2. Materials and Methods

Study Design, Setting, and Population: this prospective, observational, multicenter study was approved by the Ethics Research Committees of the hospitals where subjects were recruited. The study protocol was approved by Jerez University Hospital on 21 March 2013 (Record number 3/2013), and was subsequently approved by all the participating hospitals. All patients or next of kin signed an informed consent form. The authors complied with the World Medical Association Declaration of Helsinki regarding the ethical conduct of research involving human subjects.

We included patients admitted to the ICUs of six hospitals in Spain diagnosed with sepsis due to confirmed influenza virus A (H1N1) pneumonia over five years, from February 2013 to March 2018. Patients with other influenza A serotypes (H3N2) or influenza B were excluded, as were patients under 18 or those with incurable cancer. Patients were admitted to the ICU and data were compared with a control group (CG) of 38 patients who presented less severe influenza A H1N1pmd09v pneumonia and were not admitted to the ICU.

Criteria and definitions: Organ dysfunction was defined according to the 2001 International Sepsis Definition Conference criteria [20]. Pneumonia due to the 2009 Influenza A (H1N1) virus was defined as an infection of the lower respiratory tract characterized by clinical signs and symptoms of respiratory infection and radiologic opacities observed on chest X-ray, compatible with pneumonia [21], and with a positive respiratory sample for the influenza virus diagnostic test. ICU admission criteria (ATS/IDSA severity criteria) were as follows: major criteria (presence of one criterion) include need for mechanical ventilation or presence of septic shock; minor criteria (presence of two or more criteria), include systolic blood pressure < 90 mm Hg, respiratory rate > 30 rpm, PaO_2_/FiO_2_ < 250, multilobar infiltrators, confusion, urea > 55 mg/dL, hypothermia, leukopenia, or thrombocytopenia [21,22].

Measurements: demographic, epidemiological, clinical, and laboratory data were collected. Biomarker levels (MR-proADM, CRP, PCT, ferritin) were determined within 24 h of admission to the hospital in all patients: those admitted to the ICU (case) and not admitted to the ICU (controls).

Basic studies, including full blood count, biochemistry, and coagulation, were processed at the time of admission. Serology for atypical pneumonia, antigenuria, bronchoalveolar secretions (BAS) culture, and blood cultures, as well as nasal swabs for rapid influenza diagnostic test (Alere™ Influenza A&B test), were performed. If a false negative result of the rapid influenza diagnostic test was suspected, a real-time reverse-transcription polymerase chain reaction analysis for influenza was performed to disclose the diagnosis of influenza A (H1N1pdm) infection. Samples were collected from nasopharyngeal swab, tracheal aspirates, or BAL, if feasible, in those patients with manifestations consistent with influenza over influenza epidemic periods.

After performing full blood count and lymphocyte subpopulation assessment, the plasma was removed from the ethylenediaminetetraacetic acid (EDTA) tube by centrifugation and frozen at –80° until the MR-proADM determinations were carried out. Plasma MR-proADM was determined by immunofluorescent assay, using an automated sandwich-type immunoanalysis (Thermo Scientific™ BRAHMS™ MR-proADMKRYPTOR™; Hennigsdorf, Germany) with TRACE (Time-resolved amplified cryptate emission) technology [23].

Data Analysis: descriptive analysis of the variables was performed. The qualitative variables were expressed using a number and a percentage, while the quantitative variables were expressed using measures of central tendency (mean, median, and standard deviation). Normality was studied using the Kolmogorov–Smirnov test and homogeneity of variance was analyzed with Levene’s test. In the case of parametric quantitative variables, the two means were compared using the Student’s *t*-test; in the case of non-parametric variables, they were compared using the Mann–Whitney U test. Independence between random variables was tested using the Chi-squared test and, if the requirements for this test were not met and the variable was binary, the Fisher exact test was used. The predictive diagnostic values for sepsis and mortality were evaluated using receiver operating characteristic (ROC) curve analysis at admission. Sensitivity, specificity, predictive values, probability quotients (LR), and the area under the curve (AUC) were calculated. Mean survival was estimated using the Kaplan–Meier method. Cox proportional hazards models were created using the backward method, removing the non-significant variables from the model. Dependent variables were mortality rate in the ICU and at 90 days, and independent variables were those with a *p* value < 0.10 in the bivariate analysis; the *p* values were calculated as the corresponding hazard ratios (HR) and regression coefficients. A level of significance of 95% (*p* < 0.05) was considered in all cases. The Statistical Package for the Social Sciences (SPSS) version 22.0(SPSS Inc, Armonk, NY, USA) was used for data analysis.

## 3. Results

One hundred thirteen patients with confirmed influenza virus A (H1N1pdm09) were enrolled: 75 with severe pneumonia caused by influenza A H1N1pdm09 virus (H1N1vIPN) admitted to the ICU and 38 with less severe pneumonia admitted to the hospital or discharged from the emergency room, who were included in the control group (CG). All patients were diagnosed with influenza A (N1H1) pdm09 infection. The ICU mortality rate was 29.3%.

Treatment was protocolized according to recommendations from the Spanish Society of Intensive Medicine) (http://privada.semicyuc.org/sites/default/files/protocolo_manejo_20091015.pdf; accessed on 15 March 2013). Patients with high suspicion of influenza infection were treated with oseltamivir, 75 mg oral, twice daily. Due to the suspicion of bacterial coinfection, broad-spectrum antibiotic therapy was added until negative cultures and normal PCT were discarded. The most common antibiotic prescription was ceftriaxone.

The H1N1vIPN group (50.7% women) had a mean age of 53 (IQR 44–64) (43% under 50 and 68% under 60). The mean SOFA score was 8 (8.5–10.7), mean APACHE II was 17 (55–64), and mean SAPS II 45 (27–56). At ICU admission, the H1N1vIPN group had a significant oxygenation disorder with a median Pa/FiO_2_ ratio of 90 (60–146) mmHg. The survivors in the H1N1vIPNgroup showed greater Pa/FiO_2_ ratios with a median of 107 (72–178) vs. 55 (46–90) mmHg (*p* < 0.05). The suspicion of coinfection was 33.3% (25/75) due to the symptoms and increased biomarkers (PCT). In 11 patients, it was not possible to isolate microorganisms, whereas they were microbiologically confirmed in 13 (17.3%), predominantly gram-positives. In 17 patients, superinfection was diagnosed, predominantly gram-negatives. Regarding viruses, no case of coinfection or viral superinfection was found. Five patients had a history of hematologic malignancy (leukemia, lymphoma, and myeloma), of which two were undergoing chemotherapy during influenza infection, and they were considered immunosuppressed. Twenty-one patients (28%) had a history of immunosuppression: acquired immune deficiency syndrome (AIDS), treatment with corticosteroids or immune-suppressive drugs, or severe malnutrition. The demographic, clinical characteristics, and microbiological results at admission are shown in Table 1 and Table 2.

**Figure 1:**
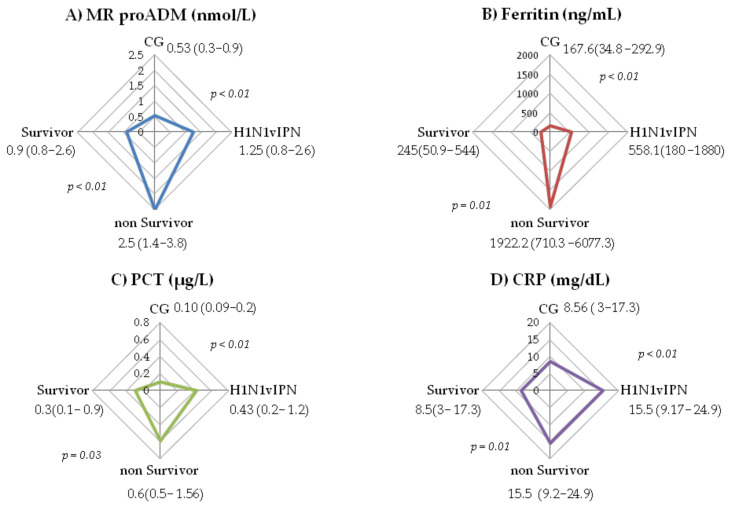
(**A**) Comparison MR-proADM levels of severity subgroups (CG vs. H1N1vIPN) and survival subgroups (non survivor vs. survivor). (**B**) Comparison ferritin levels of severity subgroups and survival subgroups; (**C**) Comparison PCT levels of severity subgroups and survival subgroups (**D**) Comparison CRP levels of severity subgroups and survival subgroups. Data are presented as median (IQR).

We compared biomarkers levels in both groups, H1N1vIPN and CG, at hospital admission. The PCT levels were 0.4 μg/L (IQR 0.2–1.2) in the H1N1vIPN group and 0.10 μg/L (IQR 0.09–0.2) in the CG (*p* < 0.01). CRP levels at admission were 15.5 mg/dL (IQR 9.2–24.9) in H1N1vIPN and 8.6 mg/dL(IQR 3–17.3) in the CG (*p* < 0.01). Ferritin levels at admission were 558.1 ng/mL(IQR 180–1880) in H1N1vIPN and 167.7 ng/mL(IQR 34.8–292.9) in the CG (*p* < 0.01). MR-proADM levels at admission were 1.3 nmol/L (IQR 0.8–2.6) in H1N1vIPN and 0.5 nmol/L (IQR 0.3–0.9) in the CG (*p* < 0.01). (Figure 1).

In the survival subgroups, all the biomarkers showed significant differences. Levels of CRP were 15.5 mg/dL (9.2–24.9) in the non-survivors and 8.5 mg/dL (3–17.3) in the survivors (*p* = 0.01). PCT levels were 0.6 µg/dL (0.5–1.6) in the non-survivors and 0.3 µg/dL (0.1–0.9) in the survivors (*p* = 0.03). Ferritin levels were 1922.2 ng/mL (710.3–6077.3) in the non-survivors and 245 ng/mL (50.9–544.1) in the survivors (*p* < 0.01). The non-survivors in the H1N1vIPN group had higher MR-proADM levels with a median of 2.5 nmol/L (IQR 1.4–3.8) vs. 0.9 nmol/L (IQR 0.8–2.6) in the survivors (*p* < 0.01). (Figure 1).

**Figure 2:**
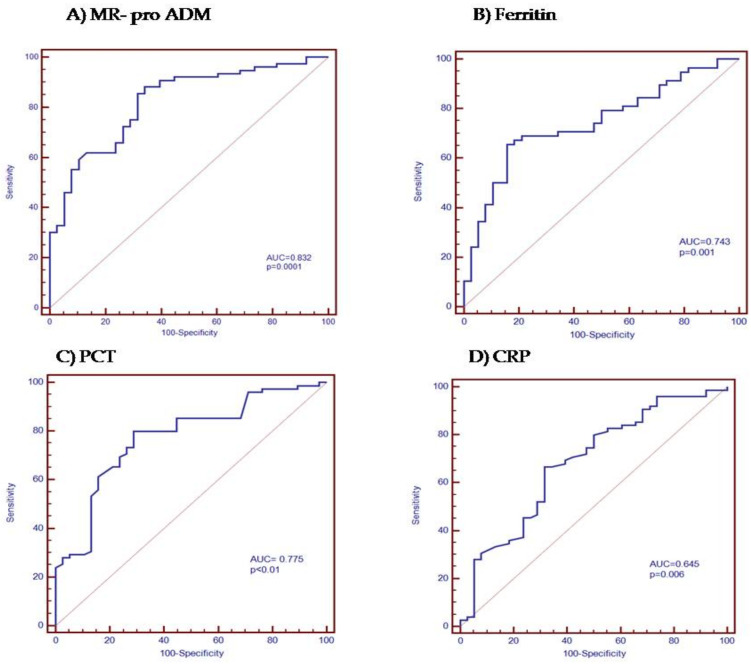
Analysis of the receiver operating characteristic curve for (**A**) MR-proADM, (**B**) ferritin, (**C**) PCT, and (**D**) CRP levels in order to predict the ICU admission.

A ROC curve analysis was performed to study the prognostic value of the different biomarkers regarding severity (admission to the ICU) and mortality. We found that the area under the curve for MR-proADM was the highest, 0.832 with a cut-off point > 1.1 nmol/L, followed by PCT (0.775) and CRP (0.645) for the prediction of ICU admission. The area under the curve for ferritin was 0.743 with a cut-off point > 345.2 ng/mL (Figure 2A–D and Table 3).

**Figure 3:**
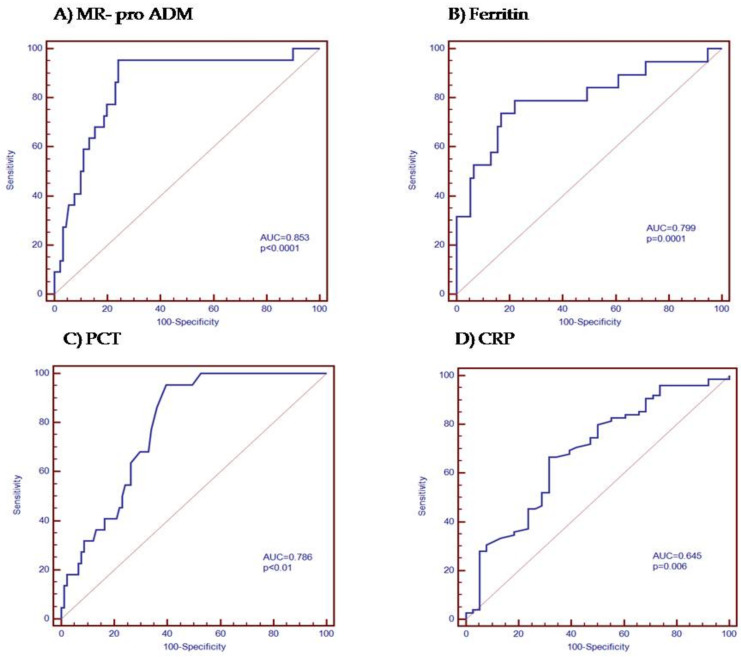
Analysis of the receiver operating characteristic curve for (**A**) MR-proADM, (**B**) ferritin, (**C**) PCT, and (**D**) CRP levels in order to predict the mortality. The prognostic value for mortality of (**A**) MR pro-ADM, ferritin, PCT, and CRP levels on admission. PPV = positive predictive value, NPV = negative predictive value.

The ROC curve analysis studying the prognostic value for mortality of MR-proADM levels on admission showed the following parameters. The AUC-ROC curve for prognostic mortality for MR-proADM was 0.853 (*p* < 0.0001), for PCT 0.786 (*p* < 0.0001), for CRP 0.696 (*p =* 0.006), and for ferritin 0.799 (*p* < 0.01). The optimal cut-off point for MR-proADM levels for predicting mortality was ≥1.2 nmol/L, with a sensitivity of 95% and a specificity of 75.8%. (Figure 3A–D and Table 4).

**Figure 4:**
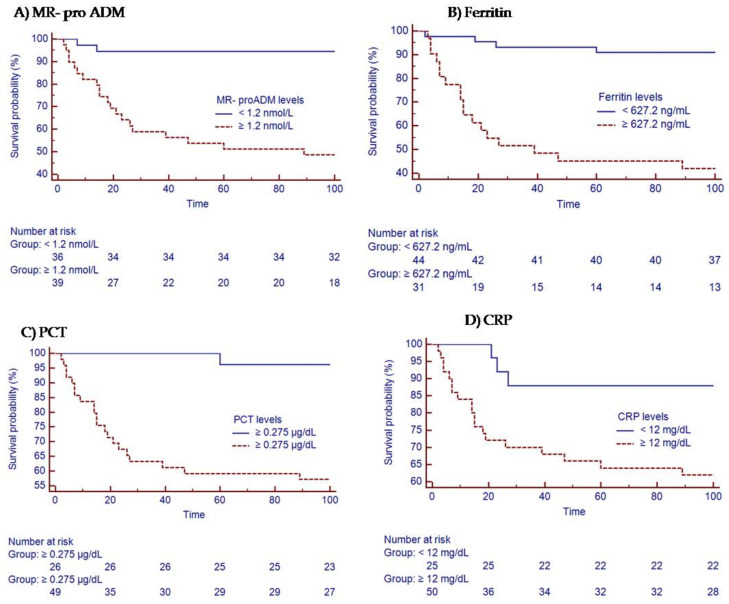
Kaplan–Meier analysis for mortality prediction at 90 days. Stratification of groups patients with severe influenza A H1N1pdm09 virus pneumonia admitted to the ICU (**A**) with MR-proADM levels greater or less than 1.20 nmol/L; (**B**) with ferritin levels greater or less than 627.2 ng/mL (**B**); (**C**) with PCT levels greater or less than 0.275 µg/dL; (**D**) with CRP levels greater or less than 12 mg/dL at admission in ICU.

Analysis of Kaplan–Meier survival curves showed that patients with MR-proADM levels of 1.2 nmol/L or above on hospital admission had significantly increased ICU and 90-day mortality (*p* < 0.01) (Figure 4A). On the other hand, ferritin levels ≥ 627 ng/mL, PCT levels ≥ 0.275 µg/dL, and CRP levels ≥ 12 mg/dL also showed a significant increase in mortality (*p* < 0.01). (Figure 4A–D and Table 4).

In the univariate analysis, a statistically significant link was found between risk of mortality in the ICU and at 90 days following admission and the following variables: APACHE II score, SOFA score, CRP, PCT, and MR-proADM levels at admission. In the multivariate analysis with Cox’s regression model performed at ICU admission, PCT (HR: 1.05; 95% CI: 1–1.1) and MR-proADM levels (HR 1.3; 95% CI: 1.1–1.6) were statistically significant factors as predictors of ICU mortality and only MR-proADM levels (HR: 1.3; 95% CI: 1.1–1.5) for 90-day mortality.

## 4. Discussion

MR-proADM levels for influenza A infections as the source of sepsis and septic shock have not been studied to date. This is the first study to report that MR-proADM plasma levels in patients assisted in the emergency room with influenza A virus pneumonia can predict disease severity, unfavorable outcome, risk of ICU admission, need for mechanical ventilation, and mortality. Moreover, we have demonstrated the prognostic superiority of MR-proADM levels over other markers such as CRP, PCT, and ferritin, and also over the severity scoring systems SOFA and Apache II.

In our series, H1N1vIPN presented the characteristic profile described in other publications [24,25]. The mean age was 53 (IQR 44–64) years; 43% of patients were under 50 and 68% were under 60. The incidence of obesity (BMI > 30) was 32.4% overall and 39.4% in the mortality group. Of the 21 patients with a history of immunosuppression, such as acquired immune deficiency syndrome (AIDS), treatment with corticosteroids, chemotherapy, or severe malnutrition, 16 evolved favorably, as did the two hematologic malignancy patients considered immunosuppressed (myeloma and leukemia). Our data support the notion that an individual history of being immunocompromised, treatment with immunosuppressive drugs, hematologic diseases, or AIDS is not related to a poor prognosis of H1N1vIPN [2,26].

The usefulness of MR-proADM in septic patients, regardless of the organism, has been demonstrated, but it has not been specifically studied in severe influenza infections. We reported that MR-proADM plasma levels measured in the ED in adults with H1N1vIPN without bacterial coinfection wereable to predict the risk of mortality and need for mechanical ventilation [27,28].

In general, there are few references to the utility of this marker in viral infections. In a study of 326 septic patients involving 20 viral infections, Andaluz-Ojeda [29] reported lower levels of MR-proADM in this subgroup compared to bacterial origin, with a median level of 1.2 nmol/L. Viral infections resulted in a milder disease severity, with a mean SOFA of 6.5, compared with 9 in patients of another origin. MR-proADM showed the best AUC-ROC for mortality prediction at 28 days, and its accuracy to predict mortality was not affected by the degree of organ failure [29]. Our series features an average SOFA of 8 with a range of 8.5–10.7. A significant number of patients at ICU admission were hemodynamically stable, with no need for vasoactive drugs and normal lactate levels, and with only respiratory failure and a moderately low SOFA score at admission. Subsequently, a high number of patients developed septic shock and required vasoactive drugs.

Moreover, the emergence of the SARS-CoV-2 pandemic during the last two years has led to the interest in relating the levels of the biomarker with the course and severity of the viral disease, taking into account their relationship with the function of the endothelium. The importance of endothelial dysfunction in the development and severity of the SARS-CoV-2 disease has been demonstrated, considering that it is a systemic disease that causes generalized endothelial damage with progressive evolution to organ failure [30,31,32,33]. On the other hand, the ADM is present in an important way in the capillary endothelium, where it maintains muscle relaxation, perfusion, and permeability, being responsible for the integrity of the capillary [33,34]. Our results are similar to those published in the last two years in SARS-CoV-2 patients, where several medium-size studies have unanimously defined MR-proADM as a marker with the ability to predict mortality [35,36,37], admission to the ICU [36,38], the appearance of ARDS [39], and the need for intubation and mechanical ventilation [36,39] or renal replacement techniques [40] in patients with COVID-19. These 12 studies add up to a total of 1316 patients. In the largest, García Guadiana et al. [36] included 356 patients with an ICU admission of 29.5% and a mortality rate of 8.9%. The MR-proADM values predicted mortality at 90 days with an AUC-ROC of 0.832. The optimal cutoff was 0.8 nmol/L with a sensitivity of 96.9%, a specificity of 58.4%, and a negative predictive value of 99.5%. In the multivariate analysis, it independently predicted mortality at 90 days (HR: 0.162). The assessment of these studies shows their usefulness in deciding the care of patients in the ED, avoiding unnecessary hospital admissions or inappropriate discharge, and assessing the escalation or reduction of support measures during the evolution of the infection, including support treatment or admission to the hospital ICU [41,42,43].

In our series, values above 1.1 nmol/L at hospital admission defined patients who developed severe respiratory failure. A high proportion of these patients will require mechanical ventilation. Values above 1.2 nmol/L are useful in defining poor prognosis in patients admitted to the ICU with a sensitivity of 100%. This cut-off point detects mortality with 100% sensitivity and a negative predictive value.

According to the literature [10,44], there is a difference in behavior between patients admitted to the ICU and the mortality groups—who reached significantly higher levels of CRP, PCT, and ferritin—those admitted to the ICU, and those who died. Interestingly, in a preliminary study [27], we reported that PCT levels measured in the emergency department (ED) in adults with H1N1vIPN without bacterial coinfection were similar in both groups (CG vs. H1N1vIPN) and showed no significant differences in mortality groups. As we know, PCT is considered a sensitive marker for bacterial infections and could be the reason why, in this case, there is a difference between risk groups due to a higher rate of coinfection at admission in severe patients; the prevalence of coinfection in the ICU group was of 33%, and reached 41% in the non-survivors. Similarly, when we analyzed an extra influenza B group with an extremely high incidence of coinfection (57.4%), PCT levels were even higher. Therefore, we can conclude that PCT measurement may help discriminate between severe lower respiratory tract infections of bacterial and H1N1vI origin. In patients admitted to the ICU with H1N1vIPN, PCT is a sensitive marker with a good negative predictive value for detecting bacterial infection and is superior to CRP. Low PCT values, particularly when combined with low CRP levels, suggest the absence of bacterial infection, either alone or in combination with influenza [10,45,46].

On the other hand, iron metabolism alteration and its relationship with the prognosis in critically ill patients is known. There is growing evidence that ferritin has an active role in modulating immunity and inflammation. It involves a physiological and beneficial response for the patient, but on certain occasions, it can reflect an excessive inflammatory response that contributes to the severity of sepsis and, therefore, to its poor prognosis [47,48]. In the case of our series, its relationship with severity and mortality is clear. The ROC curve analysis showed an AUC of 0.848 with a sensitivity of 80% and specificity of 83.5% to predict mortality, as well as in the Kaplan–Meier survival curve analysis, where values > 830 ng/mL showed a significant increase in mortality. In the multivariate analysis, ferritin levels at admission were also predictors of mortality.

The external validity of the data from this study, carried out at six public hospitals in Spain, should now be tested. If they are validated, MR-proADM may well be a good candidate for incorporation into the protocols of emergency departments for deciding on ICU admission and early treatment initiation.

Some limitations of the study should be mentioned, with the main one being the small sample size. However, the statistical power was sufficient to test the initial hypothesis, that is, the utility of baseline values of MR-proADM for predicting intensive care admission and mortality. In some patients, bacterial co-infection may confound findings because bacteria increase inflammatory biomarkers, but microbiologically confirmed co-infection was identified in only a small group (17.3%) of the cohort. In any case, a validation group should be assessed in the future.

Our results may have implications for clinical practice and research. MR-proADM levels may provide a straightforward, rapid, new avenue for improving forecast accuracy. This is particularly important in patients who only present organic respiratory failure and in whom it is difficult to predict the clinical evolution. At present, some may even be discharged home, only to return to the hospital for ICU admission and urgent respiratory support. Early warning of the likelihood of severe respiratory failure in patients with H1N1vIPN can help to guide decisions regarding the most appropriate treatment strategy and may reduce ICU mortality. MR-proADM stratifies high-risk patients adequately and is likely to benefit new treatments in patients with H1N1vIPN.

## 5. Conclusions

Initial plasma levels of MR-proADM, CRP, PCT, and ferritin effectively determine the unfavorable outcome, the risk of admission to the ICU, and mortality in patients with influenza virus pneumonia, with MR-proADM being the one that has greater predictive power for both severity and mortality.

## Figures and Tables

**Table 1:** Demographic and clinical characteristics of patients with severe pneumonia caused by influenza A H1N1pdm09 virus. Comparison based on mortality in the Intensive Care Unit.

Variable	H1N1vIPN *n* = 75	H1N1vIPN Survivors; *n* = 53; (70.67%)	H1N1vIPN Non-Survivors; *n* = 22; (29.33%)
Age median (IQR)	53 (44–64)	52 (42.7–64)	55 (47–70)
Men (%)	49.3%	49.1%	50%
Women (%)	50.7%	50.9%	50% *
BMI median (IQR)	27 (23–32)	27 (24–33.3)	27 (23–30.3)
Obesity (BMI > 30) (%)	31%	33.3%	26.3%
Smoker/COPD (%)	32.4%	34.6%	27.3%
Diabetes Mellitus (%)	21.3%	20.8%	22.7%
Immunosuppression (%)	28%	30.2%	22.7%
Hematologic malignancy(%)	6.6%	5.6 %	9%
Apache II median (IQR)	17 (12–23)	15 (12–21.7)	21 (18–25) *
SAPS II median (IQR)	45 (27–56)	37 (27–51)	53.5 (45–60) *
SOFA median (IQR)	8 (8.5–10.7)	6 (4–9)	11 (8–12) *
Lactic acid (mmol/L) median (IQR)	1.43 (0.98–1.8)	6 (4–9)	1.6 (1–2.5)
Stay in ICU median (IQR)	13 (7–23)	11 (7–23)	14.5 (7–26)
Mechanical ventilation (%)	85.3%	75%	100% *
days in MV median (IQR)	7.5 (4–17)	7 (3–16.2)	12 (5.7–22.5) *
PaO2/FiO_2_ median (IQR)	90 (60–146)	107 (72–178)	55 (46–90) *
Prone position (%)	60.3%	48.7%	84.2% *
Recruitment maneuvers (%)	27.6%	20.5%	42.1%
Septic shock (%)	56%	45.3%	81.8% *
Corticosteroids (%)	33.3%	45.3%	31.8%
Diuretics (%)	34.5%	33.3%	36.8%
Continuous renal replacement therapy (CRRT) (%)	10.7%	5.7%	22.7% *

Data are presented as median (IQR); the values expressed in percentages (%) indicate the proportion in the total group, in the survivors, and non-survivors subgroups. * *p* < 0.05 (comparison of surviving vs. non-surviving groups).

**Table 2:** List of microorganisms isolated by culture in patients with influenza A H1N1 admitted to the ICU.

Coinfection and Superinfection in Patients with Influenza A H1N1 Admitted to the ICU			
COINFECTION		SUPERINFECTION	
MICROORGANISMS	*n*. isolated cases	MICROORGANISMS	*n*. isolated cases
*Streptococcus pneumoniae*	6	*Krebsiella pneumoniae*	4
*Staphylococcus epidermidis*	2	*Staphylococcus epidermidis*	3
*Krebsiella pneumoniae*	2	*Enterococcus faecium*	3
*Candida albicans*	2	*Candida albicans*	2
*Staphylococcus aureus*	1	*Acinetobacter baumanii*	2
		*Pseudomonas aeruginosa*	2
		*Serratia marcescens*	1
Total	13	Total	17
Gram+	9	Gram+	6
Gram−	2	Gram−	9
Yeast	2	Yeast	2

**Table 3:** Specifies the AUC, Sensitivity, Specificity, PPV, NPV and optimal cut-off point for each of the biomarkers.

	AUC	Sensitivity	Specificity	PPV	NPV	Criterion
MR pro ADM	0.832	55%	90%	91.5%	50.7%	>1.1
Ferritin	0.743	65.5%	84.2%	85.4%	62.7%	>325.2
PCT	0.775	80%	71.05%	84.5%	64.3%	>0.20
CRP	0.645	66.7%	68.42%	80.6%	51%	>12

PPV = positive predictive value, NPV = negative predictive value.

**Table 4:** Specifies the AUC, Sensitivity, Specificity, PPV, NPV and optimal cut-off point for each of the biomarkers.

	AUC	Sensitivity	Specificity	PPV	NPV	Criterion
MR-proADM	0.853	95.45%	75.82%	48.8%	98.6%	>1.2
Ferritin	0.799	78.95%	77.92%	46.9%	93.7%	>627.2
PCT	0.786	95.45%	60.44%	36.8%	98.2%	>0.275
CRP	0.645	86.36%	52.75%	30.6%	94.1%	>12

PPV = positive predictive value, NPV = negative predictive value.

## Abbreviations

AIDS = acquired immune deficiency syndrome; APACHE II = acute physiology and chronic health evaluation II; AUC-ROC = area under the curve—receiver operating characteristic; COPD = chronic obstructive pulmonary disease; CRP = C reactive protein; CRRT = continuous renal replacement therapy; FiO_2_ = fraction of inspired oxygen; H1N1pdm09 = influenza virus A; H1N1vIPN = severe pneumonia caused by influenza A; ED = Emergency Department; ICU = intensive care unit; IQR = interquartile range; MR-proADM = MR-proadrenomedullin; MV = mechanical ventilation; PaO2 = arterial partial pressure of oxygen; PCT = procalcitonin; PSI = Pneumonia Severity Index; SAPS II = simplified acute physiology score; SD = standard deviation; SOFA = sequential organ failure assessment.
